# High tidal volume mechanical ventilation exacerbates pulmonary injury via upregulation of PAI-1 expression in rats

**DOI:** 10.1186/s41065-025-00446-z

**Published:** 2025-07-14

**Authors:** Jun-ming Ren, Gui-fei Wang, Jing Bi, Zhi Wang, Wei-wei Zhang

**Affiliations:** Department of Anesthesiology, Shanxi Provincial People’s Hospital, No. 29, Shuangtasi Street, Taiyuan, 030012 Shanxi China

**Keywords:** Bronchoalveolar lavage fluid, High tidal volume, Mechanical ventilation, PAI-1, Rats

## Abstract

**Objective:**

This study aimed to investigate the impact of different mechanical ventilation strategies on pulmonary plasminogen activator inhibitor-1 (PAI-1) expression in a rat model.

**Methods:**

Seventy-two specific pathogen-free (SPF) adult male Sprague-Dawley (SD) rats were randomly assigned to four groups (*n* = 18): Group C (spontaneous breathing), group S (low tidal volume, VT = 6 mL/kg), group R (regular tidal volume, VT = 10 mL/kg), and group L (high tidal volume, VT = 40 mL/kg). Each group was further divided into three subgroups based on mechanical ventilation duration (2, 4, or 6 h). Following tracheotomy intubation, group C maintained spontaneous breathing, while the other groups underwent mechanical ventilation with a small animal ventilator. Lung wet-to-dry weight ratios, cell apoptosis rate, and lung injury score were recorded. PAI-1 and IL-8 levels were measured in bronchoalveolar lavage fluid (BALF), and PAI-1 mRNA expression in lung tissue was analyzed using real-time polymerase chain reaction (PCR).

**Results:**

Progressive lung tissue injury was observed in Group L with increasing ventilation durations, accompanied by significant increases in PAI-1, IL-8, and PAI-1 mRNA expression, compared to group C (*P* < 0.05). No significant differences were identified between Group S and group C, while group R exhibited mild lung injury and minimal increases in PAI-1 expression, observed only after 6 h of ventilation. In group L, PAI-1, IL-8, and PAI-1 mRNA expression increased significantly with extended ventilation durations (*P* < 0.05).

**Conclusion:**

Mechanical ventilation strategies utilizing high tidal volumes were associated with substantial increases in PAI-1 expression in rat lung tissue and BALF, with these effects exacerbated by prolonged ventilation durations. These findings suggest that high tidal volume ventilation strategies may contribute to pulmonary injury by upregulating PAI-1 expression.

## Introduction

Mechanical ventilation is a cornerstone for managing critically ill patients and facilitating general anesthesia, however, it can lead to ventilator-induced lung injury (VILI) [1]. Extensive research, both in China and internationally, has been conducted on the pathophysiological mechanisms underlying VILI, progressing from an initial focus on mechanical injury caused by elevated airway pressure and tidal volume to insights into inflammation-induced damage [2]. However, the exact mechanisms remain partially understood due to the complex interplay of inflammatory mediators, cytokines, and signaling pathways involved in lung injury. This complexity has hindered the development of effective clinical prevention and treatment measures, resulting in persistently high morbidity and mortality rates [3]. 

Recent research has highlighted the relationship between VILI and mechanical stretch stress during high tidal volume mechanical ventilation. This stress causes direct mechanical damage and triggers changes in the morphology, structure, and membrane permeability of lung epithelial cells. These changes activate intracellular signaling pathways, promote the release of inflammatory mediators from inflammatory cells, and ultimately result in biological injury [4]. The interaction between biological and mechanical injuries further complicated VILI’s pathogenesis. Current evidence suggests that VILI involves multiple molecular mechanisms, including imbalances in pro-inflammatory and anti-inflammatory mediators, disrupted antioxidant balance, alterations in cell surface molecular conformation, cytoskeletal remodeling, and disruptions in coagulation. Additionally, genetic factors, particularly the plasminogen activator inhibitor-1 (PAI-1) gene, have also been implicated in the development of lung injury [5]. 

PAI-1 is a crucial component within the fibrinolytic system and has been implicated in the occurrence and progression of VILI. Under normal physiological conditions, cell-secreted PAI-1 regulates fibrinolysis and extracellular proteolysis [6]. Studies have shown that PAI-1 release during acute lung injury synergizes with the release of inflammatory factors. However, the specific mechanism of PAI-1 in VILI, particularly its role in the early release of inflammatory cells, remains unclear [7–9]. 

Building on previous research findings, it was hypothesized that PAI-1 is closely associated with lung injury, with increased PAI-1 secretion and expression in lung tissue corresponding to greater injury severity. Additionally, PAI-1 may influence the expression of IL-8 in inflammatory cells. Therefore, studying changes in lung PAI-1 expression under different mechanical ventilation conditions in a rat model holds practical significance. Such insights could contribute to optimizing mechanical ventilation strategies, reducing the incidence of VILI, and improving patient prognosis.

### General materials and methods

This study involved 72 healthy, specific pathogen-free (SPF) adult male Sprague-Dawley (SD) rats, each weighing between 250 and 300 g. The rats were obtained from the Beijing Xingwang Breeding Center. Experimental equipment included an animal ventilator (Chengdu TECHMAN, Model: HX-300), an animal scale (Shanghai HIKSION, Model: YB102), and an electric forced-air drying oven (Shanghai Jinghong, Model: DHG-9102). The experimental reagents included a cell apoptosis detection kit (Wuhan Boster Biological Technology Co., Ltd.), pentobarbital (Xi’an Wolsen, Batch No.: 860901), rat IL-8 and PAI-1 ELISA detection kits (Shanghai Westang Bio-Tech Inc., Ltd.), and an RT-PCR kit (Sangon Biotech (Shanghai) Co., Ltd.).

The experiment was conducted in compliance with national animal care guidelines and the ethical standards of Shanxi Medical University. The rats were housed in an environment with room temperature ranging from 20 °C to 25 °C and humidity between 40% and 70%, with 12 h of light exposure from 7:00 AM to 7:00 PM. Food and water were provided ad libitum. Prior to the experiment, rats were fasted but not water-restricted. Using a random number table, the rats were randomly assigned to one of four groups (*n* = 18): Group C (control group) spontaneous breathing, Group S (low tidal volume group) mechanical ventilation with a tidal volume (VT) of 6 ml/kg, Group R (regular tidal volume group) mechanical ventilation with a VT of 10 ml/kg, and Group L (high tidal volume group) mechanical ventilation with a VT of 40 ml/kg. Each group was further subdivided into three subgroups (*n* = 6) based on mechanical ventilation duration (2, 4, or 6 h). Group C maintained spontaneous breathing, while the other groups underwent mechanical ventilation using an animal ventilator set to the following parameters: respiratory rate (RR) of 70 breaths/min, FiO_2_ of 21%, PEEP of 0 cmH_2_O, and an inspiration-to-expiration ratio of 1:1.

### Preparation of the mechanical ventilation lung injury model

Rat lung injury models were prepared following established protocols described in previous studies [10]. After weighing, rats were anesthetized with an intraperitoneal injection of 20% pentobarbital sodium (50 mg/kg). Once anesthetized, rats were positioned on boards, and after disinfection and incision of the neck skin, the trachea was exposed. A custom-made tracheal tube was then inserted and connected to the ventilator. The neck wound was covered with warm normal saline-soaked gauze. Electrocardiographic monitoring was performed by connecting the limbs to a biological function monitor. Venous access was obtained by puncturing the tail vein, enabling the administration of fluids as necessary (1 ml/kg/h normal saline). The room temperature was maintained between 20 °C and 25 °C.

### Specimen collection

Bronchoalveolar Lavage Fluid (BALF) Collection: After successful establishment of the mechanical ventilation lung injury model, rats were euthanized by exsanguination. The thoracic cavity was accessed through the neck incision by cutting the sternum and ribs to remove the anterior chest wall. The heart and thymus were carefully separated using cotton swabs to expose the bilateral lung tissues, cervical trachea, and left and right main bronchi. A custom-made cannula was inserted 1 cm into the cervical trachea and secured with surgical sutures, while the right main bronchus was ligated. The left lung was lavaged with 2 ml of ice-cold normal saline (4 °C) through the cannula, causing lung expansion and pallor. After multiple lavages, the collected fluid was transferred to centrifuge tubes. This lavage procedure was repeated three times. The BALF was then centrifuged at 2500 r/min for 10 min, and 1.5 ml of the supernatant was stored in EP tubes at -80 °C.

Lung Tissue Sample Collection: After carefully separating the right lung tissue using cotton swabs and cutting the right main bronchus, the tracheal and fascial tissues were removed from the lung surface. Blood stains were absorbed using dry filter paper, and the lung tissue was divided into upper, middle, and lower lobes. The upper lobe was weighed and dried at 80 ℃ until a constant weight was achieved to calculate the lung wet/dry (W/D) ratio. The middle lobe was immediately stored at -80 ℃ for later testing. The lower lobe was fixed in 4% paraformaldehyde solution and sent to pathology for paraffin embedding, sectioning, and H&E staining examination.

### Index measurement and methods

1) Lung Tissue Wet-to-dry (W/D) Ratio: The upper lobe of the right lung was weighed to determine its wet weight. The sample was then placed in an electric forced-air drying oven at 80 ℃ until a constant weight was reached (dry weight). The W/D ratio was then calculated.

2) Pathological examination of lung tissue: A portion of the lower lobe of the right lung was fixed in 4% paraformaldehyde, followed by paraffin embedding, sectioning, and H&E staining. The samples were examined under an optical microscope, and lung injury was scored based on six histological features, with each feature graded on a scale from 0 to 4. The criteria were as follows: ① Alveolar wall thickening: 0 = none, 1 = mild (< 25%), 2 = moderate (25–50%), 3 = marked (> 50%), 4 = extensive confluent thickening. ② Inflammatory cell infiltration: 0 = none, 1 = few cells, 2 = multifocal aggregation, 3 = dense infiltration, 4 = diffuse infiltration. ③ Alveolar cavity bleeding: 0 = none, 1 = focal (< 10%), 2 = multifocal (10–30%), 3 = extensive (30–50%), 4 = diffuse (> 50%). ④ Interstitial edema: 0 = none, 1 = mild, 2 = moderate, 3 = marked, 4 = alveolar collapse with extensive edema. ⑤ Hyaline membrane formation: 0 = none, 1 = occasional (< 5%), 2 = localized (5–15%), 3 = multiple areas (15–30%), 4 = extensive (> 30%). ⑥ Alveolar structural destruction: 0 = intact, 1 = localized atelectasis (< 10%), 2 = multifocal (10–30%), 3 = confluent (30–50%), 4 = diffuse (> 50%). Total scores ranged from 0 to 24, with severity classified as: 0–4 (normal or mild), 5–8 (moderate), 9–12 (severe), ≥ 13 (extremely severe).

3) TUNEL Method for Lung Tissue Cell Apoptosis Detection: Following TUNEL staining, cells exhibiting brownish-yellow granules in their nuclei were considered TUNEL positive. Under high magnification (X400), five non-overlapping fields were randomly selected per section. The number of TUNEL-positive cells per 100 cells was counted to calculate the apoptotic index and its mean value.

4) ELISA (Enzyme-Linked Immunosorbent Assay) Method: This method was used to measure PAI-1 levels and inflammatory factor IL-8 expression in BALF.(Shanghai Xitang Biotechnology Co., Ltd. Batch Number: 860901).

5) The expression of PAI-1 mRNA in lung tissue was determined using RT-PCR. Total RNA was extracted from the middle lobe of the right lung tissue, which had been stored at -80 °C. The tissue was homogenized in 1 mL of Trizol reagent and incubated at room temperature for 5–10 min to separate nucleoprotein complexes from nucleic acids. Subsequently, 0.2 mL of chloroform was added, and the mixture was vigorously shaken for 15 s, followed by incubation at room temperature for 3 min. The sample was then centrifuged at 12,000 rpm at 4 °C for 10 min. The upper aqueous phase was carefully aspirated, and an equal volume of isopropanol was added. After thorough mixing, the sample was allowed to stand at room temperature for 20 min before being centrifuged to remove the supernatant. The RNA pellet was washed with 1 mL of 75% ethanol, and after centrifugation and drying, the RNA was dissolved in 30–20 µL of RNase-free ddH₂O. The extracted total RNA was reverse-transcribed into cDNA using the Taqman Reverse Transcription Kit (Shanghai Sangon Biotech Co., Ltd.), and the synthesized cDNA was stored at -20 °C for subsequent use in RT-PCR analysis.

Real-time quantitative PCR (Real-time PCR): Primer sequences:

PAI-1: Forward 5′-CTGTGACAGCCAAGAGCAAG-3′, Reverse 5′-GGTGAGACAGACGGTTGCTG-3′;

Internal reference β-actin: Forward 5′-CACCCGCGAGTACAACCTTC-3′, Reverse 5′-CCCATACCCACCATCACACC-3′.

Reaction system: Denature at 94℃ for 10 min (to activate Taq enzyme), followed by 40 cycles (95℃ for 30 s → 60℃ for 30 s → 72℃ for 30 s).

Data analysis: Read the CT value (Cycle Threshold), calculate ΔCT (target gene CT value - internal reference β-actin CT value), and further calculate the relative expression of PAI-1 mRNA through the ΔΔCT (experimental group ΔCT - control group ΔCT) and 2⁻ΔΔCT method, with the control group as the normalization baseline.

### Statistical methods

Statistical analysis was performed using SPSS 19.0 software. Measurement data were expressed as mean ± standard deviation (x̅ ± S). Intra-group and inter-group comparisons were analyzed using multivariate repeated measures analysis of variance. All data underwent tests for normality and homogeneity of variance. A *P* < 0.05 was considered statistically significant.

## Results

### Morphological changes in lungs

Macroscopic observation: Groups C and S exhibited no obvious lung tissue damage. Group R showed moderate lung tissue edema after 6 h of mechanical ventilation. Group L exhibited significant lung tissue edema and congestion after 2 h of mechanical ventilation, with numerous hemorrhagic spots appearing after 4 h, progressing to irregular patches after 6 h.

**Fig. 1:**
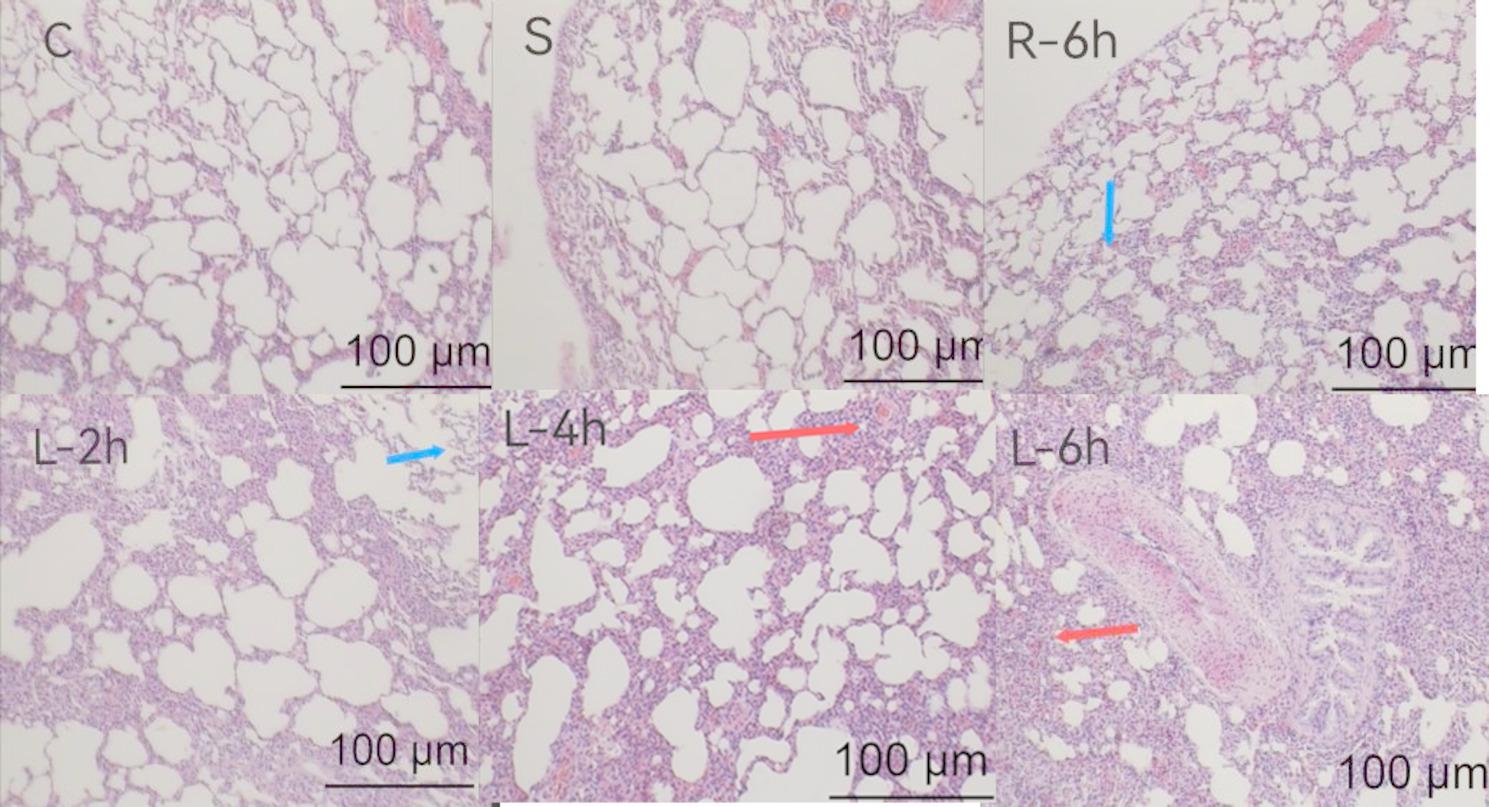
Morphological structure of rat lung tissue under an optical microscope (H&E staining, ×40 magnification) (Note: Six samples were selected for each group, and the figures shown are representative images.)

Histological Examination under Light Microscope (X40) after HE Staining: Groups C and S exhibited normal lung tissue morphology at the 2-hour, 4-hour, and 6-hour timepoints. Group R showed no significant changes at 2 h and 4 h, with minimal pulmonary exudate observed at 6 h. Group L displayed diffuse hemorrhagic spots at 2 h, followed by numerous scattered hemorrhagic spots, widened alveolar septa, and increased alveolar space exudation at 4 h. By 6 h, the lung tissue structure was completely disrupted, with alveolar fusion and tissue consolidation. The severity of lung injury in Group L increased with the duration of ventilation (see Fig. 1).

### Lung tissue W/D weight ratio, lung injury score, and apoptotic index

No significant differences were found in the W/D ratio, lung injury score, and apoptotic index between Groups C and S (*P* > 0.05). Group R exhibited no changes at the 2-hour and 4-hour timepoints compared to Groups C and S, but showed significant increases in all three indices at 6 h (*P* < 0.05). Group L displayed elevated indices starting at 2 h, significant increases at 4 h, and peak values at 6 h compared to Groups C and S. Within Group L, all three indices showed significant increases across the 2-hour, 4-hour, and 6-hour timepoints (*P* < 0.05) (see Tables 1, 2 and 3).

**Table 1:** Comparison of the lung tissue wet-to-dry weight ratios among the four groups at different time points (*n* = 6, $\:\stackrel{-}{x}$ ± S)

Groups	2 H	4 H	6 H
Group C	2.91 ± 0.42	2.83 ± 0.22	2.87 ± 0.27
Group R	2.81 ± 0.32^◇^	2.77 ± 0.47	3.40 ± 0.35^▽^
Group S	3.05 ± 0.21^◇^	3.37 ± 0.27*# ^▽^	4.13 ± 0.23*# ^▽^
Group L	4.02 ± 0.35*# ^▽^	4.67 ± 0.61*# ^△^^▽^	5.35 ± 0.58*# ^△^^▽^^◇^

Note: Comparison of groups at the same time point: *P < 0.05 vs. Group C; ^#^*P* < 0.05 vs. Group R; ^△^*P* < 0.05 vs. Group S. Comparison of time points within the same group:^▽^*P* < 0.05 vs. 2 h; ^◇^*P* < 0.05 vs. 4 h

**Table 2:** Comparison of lung injury scores among the four groups at different time points (*n* = 6, $\:\stackrel{-}{x}$ ± S)

Groups	2 H	4 H	6 H
Group C	1.33 ± 0.52	1.50 ± 0.55	1.67 ± 0.52
Group R	1.67 ± 0.52	1.83 ± 0.41	2.00 ± 0.89
Group S	2.00 ± 0.63^◇^	3.33 ± 0.52*# ^▽^	4.83 ± 0.75*# ^▽^
Group L	6.17 ± 0.75*# ^▽^	8.50 ± 1.05*# ^△^^▽^	11.5 ± 0.84*#^△^^▽^^◇^

Note: Comparison of groups at same time point: *P < 0.05 vs. Group C; ^#^*P* < 0.05 vs. Group R; ^△^*P* < 0.05 vs. Group S. Comparison of time points within same group:^▽^*P* < 0.05 vs. 2 h; ^◇^*P* < 0.05 vs. 4 h

**Table 3:** Comparison of apoptotic index among the four groups at different time points (*n* = 6, $\:\stackrel{-}{x}$ ± S)

Groups	2 H	4 H	6 H
Group C	4.17 ± 0.41	4.33 ± 0.82	4.67 ± 0.52
Group R	4.67 ± 0.82	5.33 ± 1.37	5.83 ± 0.75
Group S	4.83 ± 0.41^◇^	8.67 ± 0.82*# ^▽^	11.00 ± 0.89*# ^▽^
Group L	12.00 ± 0.89*# ^▽^	14.00 ± 1.27*#^△^^▽^	21.17 ± 1.94*#^△^^▽^^◇^

Note: Comparison of groups at same time point: *P < 0.05 vs. Group C; #*P* < 0.05 vs. Group R; ^△^*P* < 0.05 vs. Group S. Comparison of time points within same group:^▽^*P* < 0.05 vs. 2 h; ^◇^*P* < 0.05 vs. 4 h

### Changes in lung PAI-1, IL-8, and PAI-1mRNA

#### Changes in PAI-1 in BALF

Inter-group comparison: Group L exhibited significantly increased BALF PAI-1 levels with prolonged ventilation time compared to Group C at the 2-hour, 4-hour, and 6-hour timepoints (*P* < 0.05). Group R showed no changes in PAI-1 expression at 2 h and 4 h compared to Groups C and S, with an increasing trend observed at 6 h. Compared to Groups S and R, Group L showed PAI-1 expression at 2 h, marked upregulation at 4 h, and peak expression at 6 h, with significant differences (*P* < 0.05). No differences in PAI-1 expression were observed between Groups C and S.

**Fig. 2:**
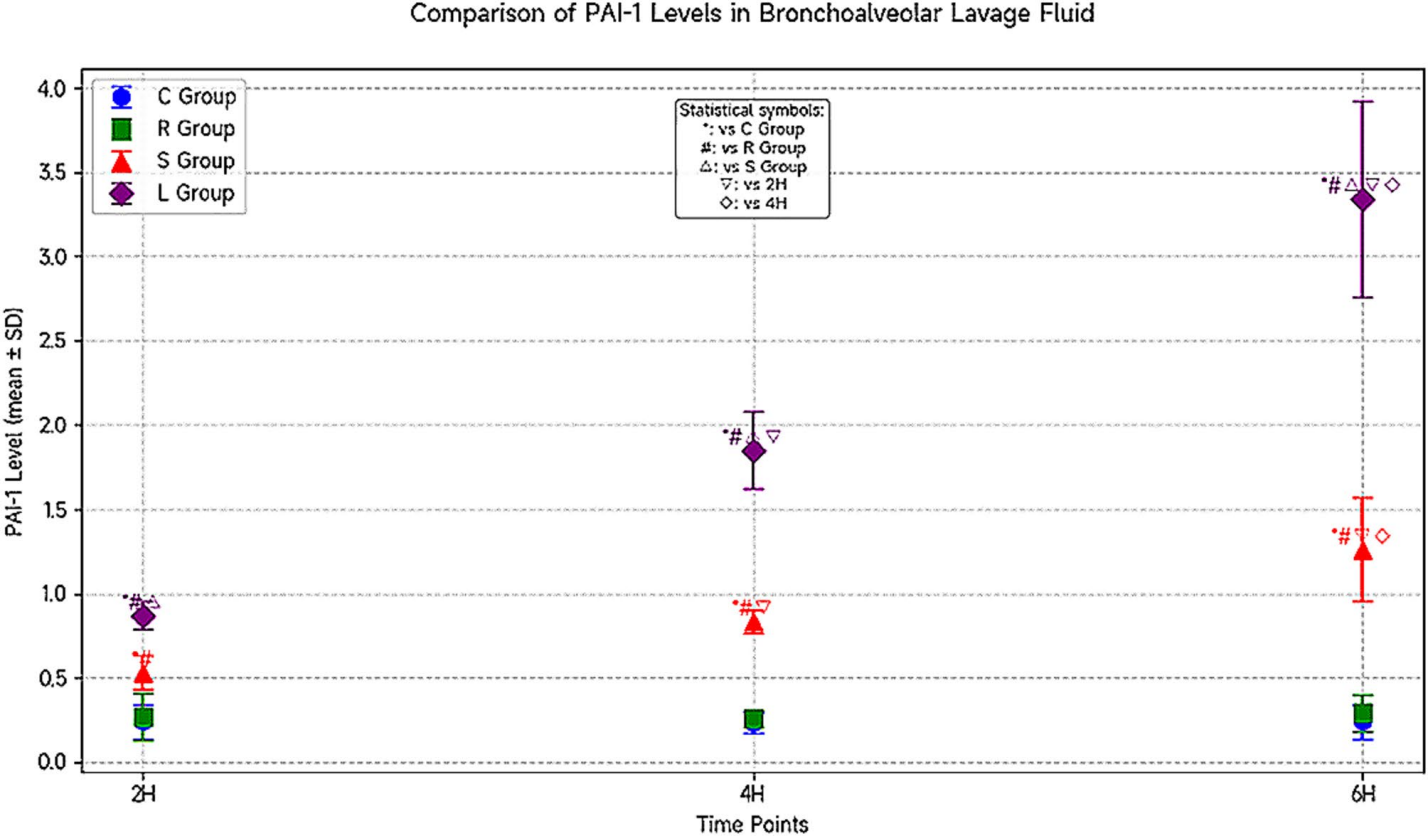
Comparison of PAI-1 levels in bronchoalveolar lavage fluid among the four groups at different time points (*n* = 6, ± S).(Shanghai Xitang Biotechnology Co., Ltd., catalog number PAI-1: WT-1001, ng/mL)

Intra-group comparison: No significant PAI-1 expression was observed at the 2-hour, 4-hour, and 6-hour timepoints within Groups C and S (*P* > 0.05). Group R exhibited minimal PAI-1 expression at 6 h. In Group L, PAI-1 expression significantly increased with the duration of mechanical ventilation (*P* < 0.05) (see Fig. 2).

#### Changes in IL-8 in BALF

Inter-group comparison: Group L exhibited significantly increased BALF IL-8 levels with prolonged ventilation time compared to Group C at the 2-hour, 4-hour, and 6-hour timepoints (*P* < 0.05). Group R showed no changes in IL-8 expression at 2 h and 4 h compared to Groups C and S, with an increasing trend observed at 6 h. Compared to Groups S and R, Group L demonstrated IL-8 expression after 2 h of mechanical ventilation, marked IL-8 upregulation at 4 h, and peak IL-8 expression at 6 h, with significant differences (*P* < 0.05). No differences in IL-8 expression were observed between Groups C and S.

**Fig. 3:**
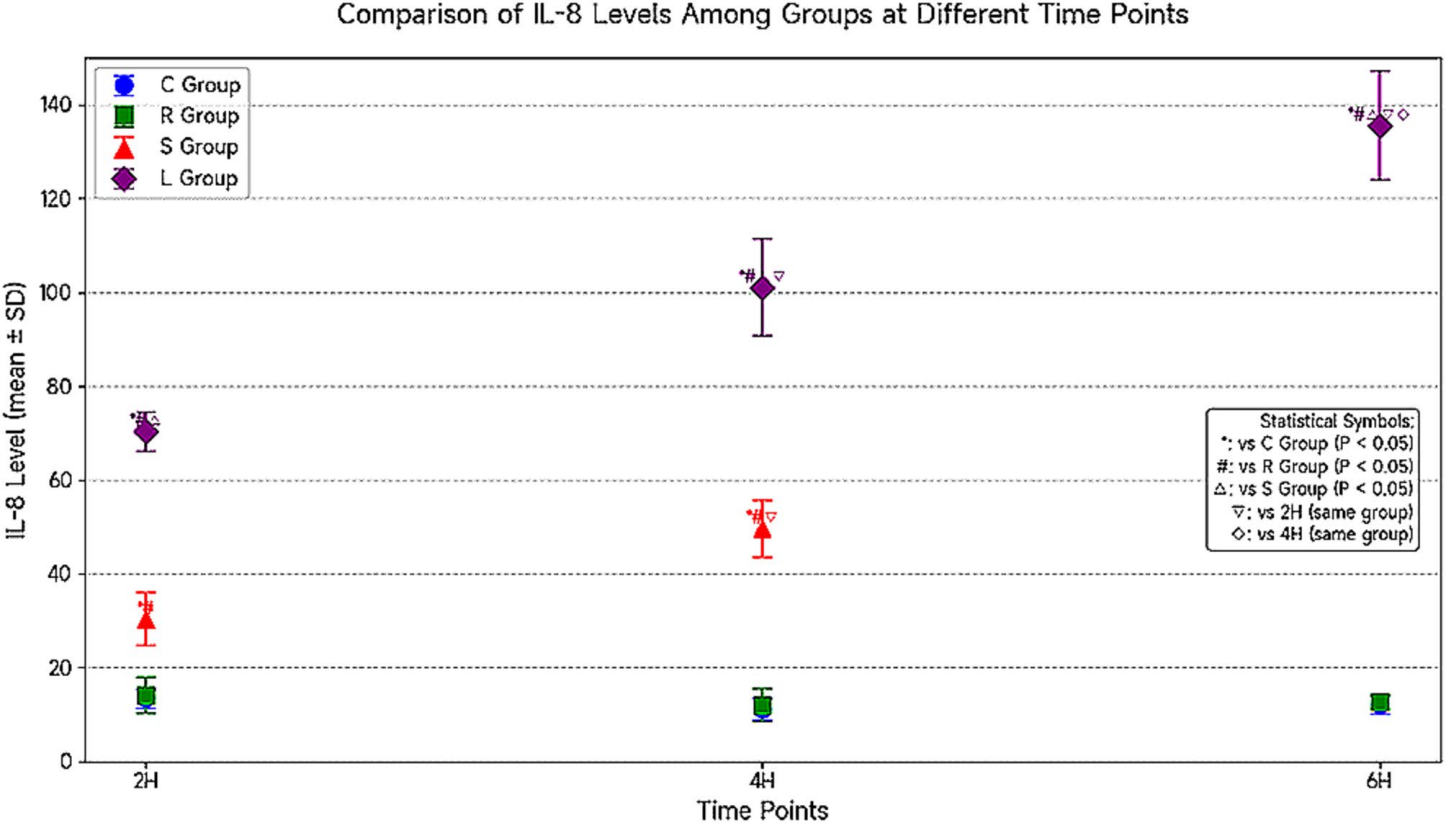
Comparison of IL-8 levels among the four groups at different time points (*n* = 6, ± S) (Shanghai Xitang Biotechnology Co., Ltd., catalog number IL-8: WT-1002, ng/mL)

Intra-group comparison: No significant IL-8 expression was observed at the 2-hour, 4-hour, and 6-hour timepoints within Groups C and S (*P* > 0.05). Group R exhibited minimal IL-8 expression at 6 h. In Group L, IL-8 expression significantly increased with the duration of mechanical ventilation (*P* < 0.05) (see Fig. 3).

#### Changes in PAI-1 mRNA expression in lung tissue

Inter-group comparison: Group L exhibited a significant increase in lung tissue PAI-mRNA expression with prolonged ventilation time compared to Group C at the 2-hour, 4-hour, and 6-hour timepoints (*P* < 0.05). Group R showed no significant changes at the 2-hour and 4-hour timepoints compared to Groups C and S but exhibited an increasing trend at 6 h. In comparison to Groups S and R, Group L showed early PAI-mRNA expression at 2 h, followed by marked upregulation at 4 h, and peak expression at 6 h, with significant differences observed (*P* < 0.05). No significant differences in PAI-mRNA expression were detected between Groups C and S.

Intra-group comparison: No significant PAI-mRNA expression was observed at the 2-hour, 4-hour, and 6-hour timepoints within Groups C and S (*P* > 0.05). Group R displayed minimal expression at 6 h. Within Group L, PAI-mRNA expression significantly increased with mechanical ventilation duration (*P* < 0.05) (see Table 4).

**Table 4:** Comparison of PAI-1 mRNA levels among the four groups at different time points (*n* = 6, $\:\stackrel{-}{x}$ ± S)

Group	2 H	4 H	6 H
C Group	0.33 ± 0.20	0.38 ± 0.16	0.32 ± 0.30
R Group	0.34 ± 0.12	0.37 ± 0.10	0.36 ± 0.30
S group	1.06 ± 0.25*#	1.42 ± 0.23*#^▽^	2.56 ± 0.99*#^▽^^◇^
L group	3.49 ± 0.49*#^△^	6.97 ± 1.26*#^△^^▽^	13.05 ± 1.55*#^△^^▽^^◇^

Note: Comparison of groups at same time point: *P < 0.05 vs. Group C; ^#^*P* < 0.05 vs. Group R; ^△^*P* < 0.05 vs. Group S. Comparison of time points within same group:^▽^*P* < 0.05 vs. 2 h; ^◇^*P* < 0.05 vs. 4 h

## Discussion

VILI, a common clinical complication, has been a major focus for research due to its complex pathogenesis. Evidence from international studies suggest that multiple mechanisms are involved, including the release of inflammatory mediators, activation of the inflammatory response, immune cell participation, changes in cellular structure and membrane permeability, and cell apoptosis or death [11, 12]. These complex biological responses often result from the conversion of mechanical stretch stimuli into intracellular signaling, with mechanosensitive genes playing a central role. When lung epithelial cells are subjected to mechanical stress, mechanosensitive genes are triggered or upregulated, initiating inflammatory cascades that ultimately lead to lung tissue injury [13]. 

In this study, the pathogenesis of VILI was investigated through mechanical ventilation models with varying tidal volumes, focusing on the roles of PAI-1 and IL-8. The findings demonstrated that high tidal volume mechanical ventilation significantly upregulated PAI-1 and IL-8 expression in lung tissue, which closely correlated with the severity of lung tissue injury. This is consistent with previous research, which showed that PAI-1, a key component of the fibrinolytic system, inhibits urokinase-type plasminogen activator (uPA) while directly regulating the inflammatory response and cell adhesion [14, 15]. In lung tissue, PAI-1 exacerbates the inflammatory response and lung tissue injury by interfering with cell adhesion and promoting neutrophil aggregation [16]. 

Our study observed that mechanical stretching significantly upregulated PAI-1 expression, although the underlying molecular mechanisms require further investigation. Recent studies suggest that mechanical stress activates PAI-1 expression through various signaling pathways. First, mechanical stretching may trigger integrin-mediated cytoskeletal remodeling, where integrins, as transmembrane receptors, convert extracellular mechanical signals into intracellular signals, activating focal adhesion kinase and the RhoA/ROCK pathway. This process promotes TGF-β1 release, which, upon binding to its receptor, enters the nucleus through Smad2/3 phosphorylation, directly binds to the PAI-1 gene promoter, and drives transcription [17]. This mechanism has been confirmed in pulmonary fibrosis models, and the sustained upregulation of PAI-1 in this study may be linked to this pathway. Second, mechanical stretching can activate the MAPK signaling pathway, including ERK1/2 and p38 MAPK. Activation of ERK1/2 phosphorylation induces the nuclear translocation of transcription factors AP-1 and NF-κB, enhancing the stability and transcriptional activity of PAI-1 mRNA [18]. The rapid increase in PAI-1 mRNA observed in the L group of this study likely reflects early activation of the MAPK pathway. These multiple pathways may contribute to the significant expression of PAI-1, but further verification of the specific regulatory pathways is needed.

Based on a rat model, our study highlighted dynamic changes in PAI-1 during VILI; however, clinical translation should be approached cautiously. Clinical studies have shown that PAI-1 levels in bronchoalveolar lavage fluid (BALF) are elevated in ARDS patients, correlating with the severity of lung injury and mortality, which aligns with the results from the L group in this study [19]. However, PAI-1 regulation in humans may be more complex. Clinical mechanical ventilation parameters, such as tidal volume and PEEP settings, are typically more conservative than those in animal models, potentially diminishing the induction of PAI-1. Additionally, human lung tissue PAI-1 expression may be influenced by genetic factors, and species-specific differences in mechanical stretch sensitivity should be considered. For instance, the homogeneity of alveolar structure in rodents makes them more susceptible to volutrauma, while the heterogeneity of human alveoli may reduce the negative effects of mechanical stress. Future studies should combine clinical cohort studies and multi-species models to further investigate the role of PAI-1 in VILI.

Notably, Groups C and S showed no significant increase in lung tissue PAI-1, IL-8, or PAI-1 mRNA expression with prolonged ventilation. This suggests that lower tidal volumes caused minimal lung tissue damage, insufficient to trigger a significant inflammatory response or upregulate PAI-1 expression. However, Group R exhibited a slight increase in PAI-1 and other inflammatory factors at 6 h, despite minimal lung tissue injury. This may be related to the rats’ baseline PAI-1 levels or could represent early signs of a mild, mechanical ventilation-induced inflammatory response [20]. 

In contrast, the high tidal volume mechanical ventilation group (Group L) exhibited significant upregulation of lung tissue PAI-1, IL-8, and PAI-1 mRNA expression at 2 h after ventilation initiation, with these increases intensifying with ventilation duration. This confirms that high tidal volume mechanical ventilation rapidly induces an inflammatory response in lung tissue. The findings also suggest that PAI-1 serves as an early and sensitive indicator of mechanical ventilation-induced lung injury [21]. By 4 h, significant lung tissue injury was observed, accompanied by elevated levels of PAI-1 and other inflammatory factors, further supporting PAI-1’s crucial role in the pathogenesis of VILI.

The findings of this study have guiding significance for the optimization of clinical mechanical ventilation strategies. First, the early upregulation of PAI-1 suggests that it can serve as a potential biomarker for VILI. In clinical practice, the levels of PAI-1 in bronchoalveolar lavage fluid or serum can be monitored to identify high-risk patients early and adjust ventilation parameters. Second, limiting tidal volume (such as adopting a protective ventilation strategy of 6 ml/kg) can significantly reduce the expression of PAI-1, which is consistent with the principle of lung-protective ventilation recommended by international guidelines [22].

However, this study has certain limitations. First, while the effects of mechanical ventilation on PAI-1 and IL-8 expression were investigated in a rat model, the applicability of these findings to human populations requires further validation [23]. Second, focusing only on PAI-1 and IL-8 provided limited insight into the complex pathogenesis of VILI, which involved multiple inflammatory factors and signaling pathways. Further studies are needed to explore additional factors and signaling pathways contributing to VILI [24]. Additionally, while significant upregulation of PAI-1 was associated with high tidal volume mechanical ventilation, the specific molecular mechanisms—including the conversion of mechanical stretch to intracellular signaling and activation of PAI-1 expression—require further investigation.

In conclusion, this study preliminarily confirmed through a rat model that mechanical ventilation can induce the upregulation of PAI-1 and IL-8 expression in lung tissue, and this upregulation is closely related to the severity of lung tissue injury. This result not only provides new clues for a deeper understanding of the pathogenesis of VILI, but also offers potential targets for future efforts to find effective means of preventing and treating VILI. With the development of emerging technologies such as artificial intelligence (AI) and machine learning, biomedical research is being completely transformed through the realization of rapid data integration and predictive modeling. For example, the AI-driven biosimulation tool demonstrated by Sánchez-Herrero et al. (such as PhysPK) can be adapted to simulate the mechanically stressed-induced molecular pathways in VILI [25]. Similarly, the AI platform for early lung cancer diagnosis and patient triage has shown how machine learning can enhance the identification of high-risk VILI patients through real-time biomarker analysis (e.g., PAI-1 levels in BALF or serum) [26]. In addition, advances in mRNA-based therapies have provided new avenues for targeting PAI-1 or IL-8 in VILI. For example, the self-assembling peptide platform studied by Wang et al. for tumor vaccines can be repurposed to deliver mRNA constructs that silence PAI-1 expression or modulate the inflammatory cascade in damaged lung tissue [27]. Our study, by identifying PAI-1 as a sensitive biomarker and mechanism-driven factor for VILI, provides a foundational framework for these technologies. In the future, AI-driven predictive models can be combined with multi-omics datasets to reveal the complex interplay between mechanical stress, PAI-1 signaling, and inflammatory responses. Through collaboration among bioengineers, data scientists, and clinicians, we hope to achieve more breakthroughs and provide new ideas and methods for the prevention and treatment of VILI.

## Conclusion

High tidal volume mechanical ventilation induces significant upregulation of PAI-1 in both lung tissue and BALF in rats. Changes in PAI-1 expression are detectable as early as 2 h after the initiation of mechanical ventilation, become pronounced at 4 h and reach their peak at 6 h. These findings underscore the role of PAI-1 as a sensitive early marker of ventilator-induced lung injury (VILI).

## Data Availability

The datasets used and/or analysed during the current study available from the corresponding author on reasonable request.

## Abbreviations

VT: Tidal volume
BALF: Bronchoalveolar lavage fluid
VILI: Ventilator-induced lung injury
W/D: Wet/dry
IL-8: Interleukin-8
PAI-1: Plasminogen activator inhibitor-1
uPA: Urokinasetypeplasminogenactivator
